# The Warwick-Edinburgh Mental Well-being Scale (WEMWBS): development and UK validation

**DOI:** 10.1186/1477-7525-5-63

**Published:** 2007-11-27

**Authors:** Ruth Tennant, Louise Hiller, Ruth Fishwick, Stephen Platt, Stephen Joseph, Scott Weich, Jane Parkinson, Jenny Secker, Sarah Stewart-Brown

**Affiliations:** 1 Warwick Medical School, University of Warwick, Coventry, UK; 2 Research Unit in Health, Behaviour and Change, School of Clinical Sciences & Community Health, University of Edinburgh, Edinburgh, UK; 3 School of Sociology & Social Policy, University of Nottingham, Nottingham, UK; 4 NHS Health Scotland, Glasgow, UK; 5 Faculty of Health and Social Care, Anglia Ruskin University, Cambridge, UK

## Abstract

**Background:**

There is increasing international interest in the concept of mental well-being and its contribution to all aspects of human life. Demand for instruments to monitor mental well-being at a population level and evaluate mental health promotion initiatives is growing. This article describes the development and validation of a new scale, comprised only of positively worded items relating to different aspects of positive mental health: the Warwick-Edinburgh Mental Well-Being Scale (WEMWBS).

**Methods:**

WEMWBS was developed by an expert panel drawing on current academic literature, qualitative research with focus groups, and psychometric testing of an existing scale. It was validated on a student and representative population sample. Content validity was assessed by reviewing the frequency of complete responses and the distribution of responses to each item. Confirmatory factor analysis was used to test the hypothesis that the scale measured a single construct. Internal consistency was assessed using Cronbach's alpha. Criterion validity was explored in terms of correlations between WEMWBS and other scales and by testing whether the scale discriminated between population groups in line with pre-specified hypotheses. Test-retest reliability was assessed at one week using intra-class correlation coefficients. Susceptibility to bias was measured using the Balanced Inventory of Desired Responding.

**Results:**

WEMWBS showed good content validity. Confirmatory factor analysis supported the single factor hypothesis. A Cronbach's alpha score of 0.89 (student sample) and 0.91 (population sample) suggests some item redundancy in the scale. WEMWBS showed high correlations with other mental health and well-being scales and lower correlations with scales measuring overall health. Its distribution was near normal and the scale did not show ceiling effects in a population sample. It discriminated between population groups in a way that is largely consistent with the results of other population surveys. Test-retest reliability at one week was high (0.83). Social desirability bias was lower or similar to that of other comparable scales.

**Conclusion:**

WEMWBS is a measure of mental well-being focusing entirely on positive aspects of mental health. As a short and psychometrically robust scale, with no ceiling effects in a population sample, it offers promise as a tool for monitoring mental well-being at a population level. Whilst WEMWBS should appeal to those evaluating mental health promotion initiatives, it is important that the scale's sensitivity to change is established before it is recommended in this context.

## Background

There is increasing international interest in the concept of positive mental health and its contribution to all aspects of human life. The World Health Organisation [1] has declared positive mental health to be the 'foundation for well-being and effective functioning for both the individual and the community' and defined it as a state 'which allows individuals to realise their abilities, cope with the normal stresses of life, work productively and fruitfully, and make a contribution to their community.' The capacity for mutually satisfying and enduring relationships is another important aspect of positive mental health [2].

The term positive mental health is often used in both policy and academic literature, interchangeably with the term mental well-being. It is a complex construct, covering both affect and psychological functioning with two distinct perspectives:- the hedonic perspective, which focuses on the subjective experience of happiness and life satisfaction, and the eudaimonic perspective, focusing on psychological functioning and self realisation [3]. These perspectives, which have informed distinct bodies of research in positive mental health, are less obvious in the literature relating to poor mental health, where items measuring affect (feeling happy/sad) are often combined with items measuring psychological functioning (playing a useful part in things, making decisions) [4] in the same scales, suggesting that poor mental health at least is accepted as involving limitations in both eudaimonic and hedonic well-being [5-7]. Positive mental health is recognised as having major consequences for health and social outcomes [8,9]. This has given rise to new positive psychological therapies that are explicitly focused on facilitating positive mental health [10-12]. However the field of positive mental health is under-researched partly because of the lack of appropriate population-based measures [13]. There is demand from those interested in public mental health for a measure suitable for monitoring mental well-being that does not show ceiling effects in population samples. There is also demand from mental health promotion practitioners for a measure with which they can evaluate their programmes. Measures with a negative focus can suggest to participants that such programmes are for people with mental health problems and in this way detract from, rather than support, these initiatives.

Existing instruments in this field take different conceptualisations of well-being as their starting point. The commonly-used twenty-item PANAS scale [14] describes affective-emotional aspects of well-being and is comprised of two dimensions: positive and negative affect (PANAS-PA and PANAS-NA) which are reported as distinct and independent concepts. In contrast, the five-item Satisfaction With Life Scale (SWLS) [15] aims to measure cognitive-evaluative facets of well-being. The 54 item Scale of Psychological Well-Being (SPWB) [16] focuses on eudaimonic well-being and assesses psychological functioning. Its sub-scales measure autonomy, self-acceptance, environmental mastery, purpose in life, personal growth and positive relations with others. The five-item Short Depression-Happiness Scale (SDHS) [17] developed for use in therapeutic settings assesses well-being as a continuum between the two states of depression and happiness. All these instruments cover aspects of mental illness as well as mental health and include positive and negatively worded items. The positively worded five item WHO Wellbeing Index (WHO-5) [18] aims to measure overall well-being and covers aspects of physical as well as mental health.

We report here on the development and testing of a new scale – the Warwick-Edinburgh Mental Well-Being Scale (WEMWBS). This scale aims to build on previous scales and capture a wide conception of well-being, including affective-emotional aspects, cognitive-evaluative dimensions and psychological functioning, in a form which is short enough to be used in population-level surveys. By focusing wholly on the positive, the scale is intended to support mental health promotion initiatives and be free of ceiling effects in population samples.

The starting point for the development of this scale was the Affectometer 2 [19], a scale developed in New Zealand in the 1980s which aimed to measure well-being and had intuitive appeal to those working in mental health promotion in the UK, because it covered both eudemonic and hedonic aspects of mental health and had a good range of positive items [20]. This scale comprised 20 statements and 20 adjectives relating to mental health in which positive and negative items are balanced. The UK validation of Affectometer 2 reported good face validity, favourable construct validity with comparable scales, good discriminatory powers between different population groups and appropriate test-retest reliability over time [21,22]. The scale also had important limitations: its very high level of internal consistency (r = 0.94) suggested redundancy, its susceptibility to social desirability bias was higher than that of other comparable scales and its length was a potential barrier to its uptake as a measure of population well-being. This study aimed to develop a new scale of mental well-being with a single underlying construct that encompassed a broad range of attributes associated with mental well-being and to validate this scale using data collected from student and population samples.

## Methods

### Participants and data collection – scale development

Nine focus groups were held, three in England and six in Scotland. Participants were recruited through community groups, selected to cover a range of attributes (age, sex, socio-economic status) that are known to be associated with mental health [23]. In addition, one focus group was carried out with mental health service users. Focus groups were made up of a maximum of eight participants, and a total of 56 people took part. Participants were asked to complete the Affectometer 2, and to discuss their concept of positive mental health and its relationship with items in this scale. All focus groups were taped and transcribed. Content analysis was used to identify items which participants across the groups found consistently confusing or difficult to understand and concepts relating to mental well-being which participants thought should be included in the scale. Full details of focus groups are reported elsewhere [21]. Factor loadings and completion rates for individual items from a general population survey were examined for each of the Affectometer 2 items [22].

### Development of WEMWBS

An expert panel representing the disciplines of psychiatry, psychology, public health, social science and health promotion with expertise in mental health and well-being was convened to consider the results of the UK validation of Affectometer 2 [21,22] and the analysis of focus group discussions. With reference to current academic literature describing psychological and subjective well-being, the expert panel agreed key concepts of mental well-being to be covered by the new scale: positive affect and psychological functioning (autonomy, competence, self acceptance, personal growth) and interpersonal relationships. Using this framework and data from the qualitative and quantitative studies described above, the panel identified items for retention and rewording from Affectometer 2 and agreed the wording of new items. A new scale composed only of positively worded items relating to aspects of positive mental health was developed [see Additional file 1].

The final scale consisted of 14 items covering both hedonic and eudaimonic aspects of mental health including positive affect (feelings of optimism, cheerfulness, relaxation), satisfying interpersonal relationships and positive functioning (energy, clear thinking, self acceptance, personal development, competence and autonomy).

Individuals completing the scale are required to tick the box that best describes their experience of each statement over the past two weeks using a 5-point Likert scale (none of the time, rarely, some of the time, often, all of the time). The Likert scale represents a score for each item from 1 to 5 respectively, giving a minimum score of 14 and maximum score of 70. All items are scored positively. The overall score for the WEMWBS is calculated by totalling the scores for each item, with equal weights. A higher WEMWBS score therefore indicates a higher level of mental well-being.

## Validation of WEWMBS

### Participants and data collection – scale validation

Quantitative data were collected from two samples. Initial scale testing was carried out using data collected from convenience samples of undergraduate and postgraduate students at Warwick and Edinburgh universities. Students were recruited from seven disciplines. Scales were administered at the end of scheduled teaching sessions. Participants were given the option of completing scale packs on the spot or in their own time and were given a pre-addressed envelope to return completed packs.

Students were asked to provide information on age, sex and subject being studied, and to complete WEMWBS and between two and four other scales each from a pool of eight different scales. Scales were assigned randomly to students, with WEWMBS either appearing at the beginning or end of the sequence of scales. To assess the scale's test-retest reliability, a random sub-sample of students who had completed the scale pack was given the WEWMBS scale to complete one week later. Students were asked to use a unique identifier on both occasions so that data collected in the first week could be matched to data collected one week later.

A second set of combined data from two representative Scottish population datasets - the 2006 September wave of the Scottish Health Education Population Survey (HEPS) [24] and the 2006 Well? What do you think? Survey [25] - was used to test the results obtained from the student sample, and to assess whether the scale discriminated between population groups in a way that was consistent with the findings of national psychiatric morbidity surveys [26].

Allowing for invalid addresses, a response rate of 66% was achieved in HEPS and 57% in the Well? What do you think? Survey, accruing 859 and 1,216 interviews respectively. Interviews were carried out face to face, in people's homes, using Computer Assisted Personal Interviewing.

NHS Health Scotland commissioned the HEPS which was carried out by BMRB International and the Scottish Executive commissioned the Well? What do you think? survey which was carried out by Ipsos MORI and Stirling University.

Statistical tests carried out on these two samples (student and population) are summarised in Table 1. Only data where WEMWBS was fully completed were used. Unweighted data were used for the population sample.

**Table 1:** Summary of psychometric tests carried out on two samples

**Psychometric property**	**Statistical test**	**Student sample (number)**	**Population sample (number)**
Content validity	Responder bias: Chi-square tests	-	2075
	Missing and popular responses	348	2075
	Floor/ceiling effects (individual items)	348	1749
Construct validity	Confirmatory Factor Analysis	348	1749
Internal consistency	Cronbach's α 's	348	1749
	Item-total score correlations	348	1749
Criterion validity	Floor and ceiling effects (total score)	348	1749
	Demographic differences in scores:	-	1749
	Wilcoxon rank sum tests/Kruskal-Wallis tests/Jonckheere's test		
	Correlations with other scales:		
	Spearman's rank correlation coefficient	72 (EQ-5D VAS)	1233 (GHQ-12)
		63 (PANAS- PA/NA)	
		63 (SPWB)	
		71 (SDHS)	
		79 (WHO-5)	
		79 (SWLS)	
		77 (GLS)	
		67 (EIS)	
	Jonckheere's test	-	1233(GHQ-12)
Reliability	Intra-class correlation coefficients	124	-
Social desirability bias	Spearman's rank correlation coefficient	116	-

### Validation measures

Eight additional scales were included in the student sample questionnaire to validate WEWMBS and one was available in the population sample. These scales were chosen to include those that measured either the same or similar concepts to WEMWBS or concepts that were expected to be associated with mental well-being such as emotional intelligence and general health. Specific prior hypotheses about the relationship between WEMWBS and each of the eight scales were developed. The scales included two covering positive and negative aspects of affect (PANAS, SDHS), one covering psychological functioning (SPWB), one overall well-being (WHO-5), two scales measuring life satisfaction (SWLS and the single-item Global Life Satisfaction scale (GLS) [27]), and one scale, the 33-item Emotional Intelligence Scale (EIS) [28] which consists of statements covering appraisal, expression, and regulation of emotion in self and others, and the utilisation of emotions in problem solving. Information about health status was assessed using the EuroQol Health Status Visual Analogue Scale (EQ-5D VAS) [29] which asks respondents to rate their overall health (physical as well as mental) on a 0–100 scale.

Data on mental ill-health was collected in the two population datasets using the GHQ-12 [4] which asks participants about their general level of happiness, experience of depressive and anxiety symptoms, and sleep disturbance over the last four weeks. Other variables of interest were collected in the two population datasets: data on sex, age, housing tenure, self-perceived health status and employment status in both the HEPS and Well? What do you think? Survey. In addition, the HEPS also collected data on marital status, gross household income, age of leaving formal education, and social grade of chief income earner.

Social desirability bias was assessed in the student sample using the Balanced Inventory of Desirable Respose (BIDR) [30] which includes sub-scales measuring impression management and self-deception.

### Content validity

The frequency of complete responses to WEMWBS from both the student and population samples was examined to assess the perceived relevance and adequacy of WEMWBS to the target population. Using data from the population sample, the demographics of complete responders were compared to those who partially or non-responded to the scale using Chi-square tests with continuity corrections and Chi-square tests for trend where appropriate.

For assessment of relevance, sensitivity and signs of inappropriateness, the incidence of missing item responses was considered. Additionally, the distributions of responses from complete responders within the student and population sample highlighted the frequency of popular responses and any floor and ceiling effects.

### Construct validity

Confirmatory factor analysis using weighted least squares estimation was undertaken on item responses from both the student and population samples to test the appropriateness of the structural equation models that specified the pre-hypothesised one-factor structure of WEMWBS. Analysis was undertaken using the SAS statistical software, initially assuming no dependencies between residuals and then with stepwise addition of the matrix element representing the highest dependency until adequate fit statistics were obtained.

The goodness of fit index (GFI) and adjusted goodness of fit index (AGFI), based on a correction for degrees of freedom, were assessed with their desired levels being > 0.9 and > 0.8 respectively [31,32]. The Root Mean Square Error of Approximation (RMSEA) was below the desired 0.06 level [33], thus indicating only a small amount of unexplained variance or residual. The chi-squared statistic, however, with a p-value < 0.05, indicates a significant amount of actual covariance between measures that was unexplained by the models [34]. However, large sample sizesmay lead to an overstatement of lack of fit [32].

### Internal consistency

Cronbach's alpha was calculated for each of the student and population samples to measure the homogeneity of the global score. Internal consistency estimates of > 0.70 were sought [35]. Additionally, to assess for item-redundancy, Cronbach's alpha was calculated for different sized reduced versions of the scale to identify at what point the Cronbach's alpha would fall to an unacceptable level. For each reduced size, 10 different choices of item components were randomly chosen and the range of Cronbach's alpha statistics was considered. For further assessment of internal consistency, item-total score correlations, adjusted for overlap, were calculated for each item; substantial but not excessive values (greater than or equal to 0.2 and less than 0.8) were sought [36].

### Criterion validity

Total and item scores were examined for floor and ceiling effects and the normality assumption investigated using the Shapiro-Wilk test on both samples.

Correlations between scores on the WEMWBS and eight other scales capturing different dimensions of physical and mental health and well-being were calculated using Spearman's rank correlation coefficients, using data from the student sample. Population sample data were used to generate Spearman's rank correlation coefficients and Jonckheere's tests for ordered alternatives as appropriate for WEMWBS scores and the scores generated from the GHQ-12 [4]. Based on the content of each scale, we hypothesised that WEMWBS would show high correlations with scales capturing positive affect or well-being (SDHS, WHO-5, PANAS-PA and SPWB) moderate correlations with scales measuring physical or mental health status (GHQ-12, EQ5D-VAS) and the PANAS-NA and lower correlations with life satisfaction scales (GLS and SWLS) and emotional intelligence (EIS).

Prior hypotheses about the expected association between WEMWBS score and factors known to predict poor mental health were developed. Based on the findings of recent U.K. psychiatric morbidity studies [23,26], we hypothesised that men would show a higher score than women, that there would be no association with age at leaving full-time education and that the scale would show a positive association with higher socio-economic status. Differences in scores across demographic groups were assessed for criterion validity using Wilcoxon rank sum tests, Kruskal-Wallis tests and Jonckheere's tests for ordered alternatives, as appropriate, using the population sample.

Social desirability bias was assessed on the basis of Spearman's rank correlation coefficients between WEMWBS and scores on the impression management sub-scales of the BIDR, using data from the student sample. For comparative purposes, correlations between the two BIDR sub-scales and four other scales, (SWLS, WHO-5, PANAS-PA and PANAS-NA, and single-item GLS) were also calculated.

### Reliability

The scale's test-retest reliability at one week was assessed, using intra-class correlation coefficients, using data collected from a sub-sample of the student sample.

### Ethics

This study was approved by Warwick Medical School's Ethics Committee. Written consent for publication was obtained from the participants.

## Results

### Response rates

In the student sample, 354 students from seven disciplines completed scale packs containing WEMWBS and between two and four other scales. The overall response rate was 53%. Of those who responded, 348 (98%) fully completed WEMWBS. In the second week of testing (test-retest reliability) 124 out of 266 (47%) students fully completed WEMWBS.

In the population sample of size 2075, 323 (16%) failed to answer any WEMWBS items and a further 3 responded only partially. Partial or non-responders were more likely to be older (p < 0.01), own their house outright or rent (p < 0.01), be in worse general health (p < 0.01), be retired (p < 0.01), have left education at an earlier age (p < 0.01) and have the chief household earner of a higher social class (p < 0.0001) than complete responders. No differences were observed according to respondents' sex (p = 0.29), marital status (p = 0.38) or household income (p = 0.30).

### Content validity

**Figure 1:**
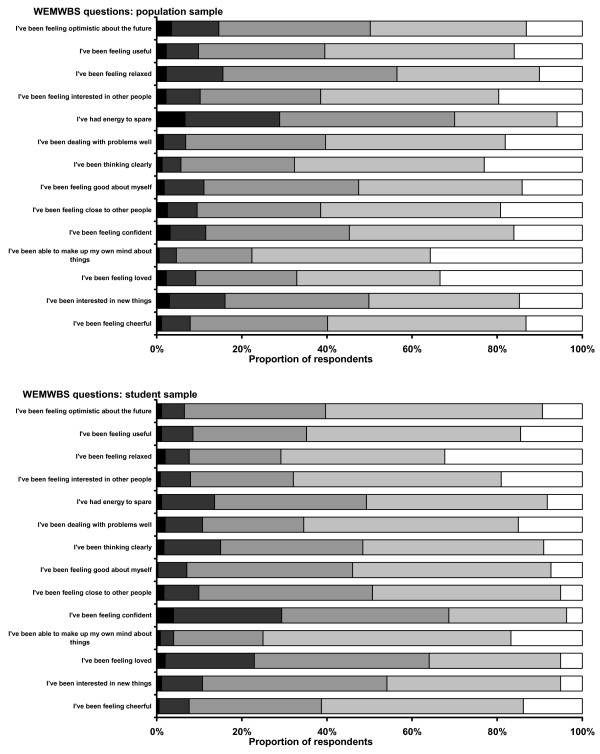
WEMBS question responses: student and population samples.

Assessment of item response frequencies from complete responders in each sample showed little evidence of highly skewed distributions, with all response categories being used by at least one person for all items (Figure 1).

### Construct validity

Confirmatory factor analysis of the 1749 respondent population sample showed the estimated factor matrix was proven to match with the hypothesized factor matrix. The GFI and AGFI were both above their desired levels (GFI = 0.91 and AGFI = 0.87). Additionally, the RMSEA = 0.0502 fell below the desired upper limit. Although the chi-squared statistic indicated a significant lack of fit, the relatively large sample size needs to be taken into consideration when interpreting this finding. Confirmatory factor analysis from the 348 respondent student sample showed adequate GFI, AGFI and RMSEA value (GFI = 0.93, AGFI = 0.89, RMSEA = 0.0551). A significant chi-squared statistic was again obtained (chi squared = 141.6, df = 69, p < 0.0001). From these results, both samples showed verification of the pre-hypothesised one-factor scale structure. For each sample, all items loaded > 0.5 onto the single factor.

### Internal consistency

**Figure 2:**
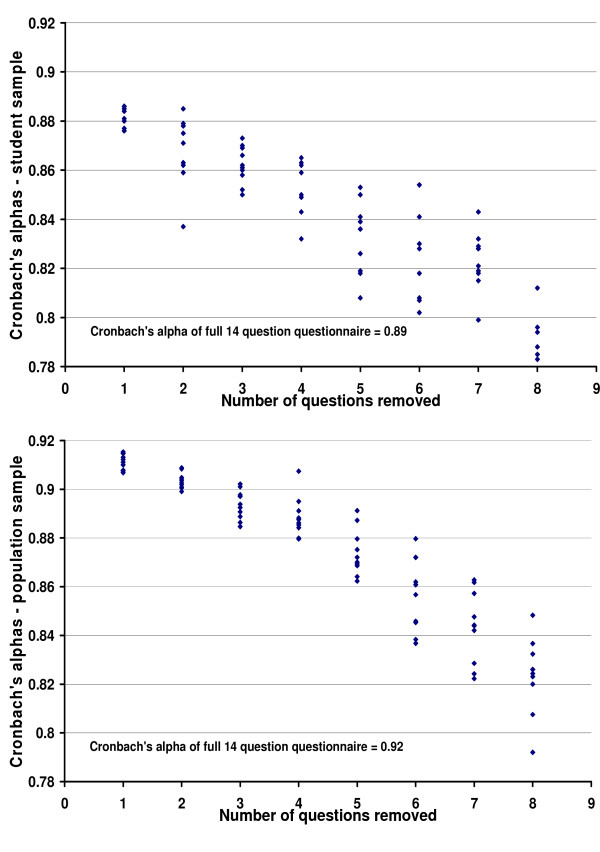
Cronbach's alphas of 10 randomly generated versions of WEMWBS: student and population samples.

The standardised Cronbach's alpha was 0.89 for the student sample and 0.91 for the population sample, falling well above the recommended lower limit. The standardised Cronbach's alphas for the 10 randomly selected reduced 13 item versions of the WEMWBS had ranges falling well above the 0.7 limit. Only when 6 items had been deleted and 8 remained did the Cronbach's alpha fall below even 0.8 for one of the 10 randomly selected versions of the scale in the student sample. Cronbach's alpha remained above this level in the population sample until 8 items had been deleted (Figure 2).

WEMWBS scores were calculated for all responders. Item-total correlations, corrected for overlap, for all items ranged between r = 0.52 and 0.80 (student sample) and r = 0.51 and 0.75 (population sample). These correlations are within the desired limits, which supports the validity of this global score.

### Criterion validity

**Figure 3:**
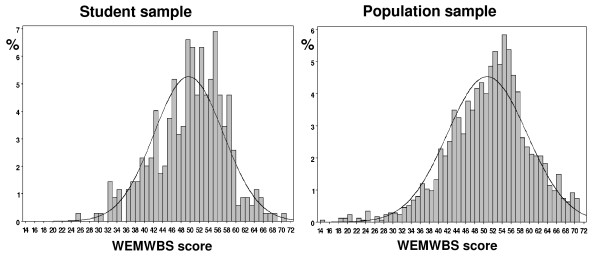
Score distribution for student and population samples.

Although scale scores were reasonably Normally distributed, results in this large population sample showed significant non-Normality (p < 0.01), with a slight negative skew. WEMWBS score did not appear to suffer from floor and ceiling effects in either sample (Figure 3).

The median score was 50 in the student sample and 51 in the population sample, with inter-quartile ranges of 45 – 55 and 45 – 56 respectively.

In the student sample, overall health, as represented by the EQ-5D VAS, showed a low to moderate significant correlation (r = 0.43, p < 0.01), as hypothesised (Table 2). Also as hypothesised, scales measuring components of affect or well-being all showed significant high correlations with WEMWBS: (PANAS-PA r = 0.71, p < 0.01, SPWB r = 0.74, p < 0.01, SDHS r = 0.73, p < 0.01, WHO-5 0.77, p < 0.01) (Table 2). A moderate negative correlation was observed between WEMWBS and the PANAS-NA (r = -0.54, p < 0.01) (Table 2). The two life satisfaction scales showed higher than anticipated correlations with WEWMBS (SWLS r = 0.73, p < 0.01, GLS 0.53, p < 0.01) (Table 2). As hypothesised, the EIS showed a low to moderate correlation with WEMWBS (r = 0.48, p < 0.01) (Table 2).

**Table 2:** Correlations between WEMWBS and other scales: student sample

**Scale**	**N**	**Correlation with WEMWBS**
Overall health		
EQ-5D VAS	72	0.43*
Well-being/affect		
PANAS- PA	63	0.71*
PANAS- NA	63	-0.54*
Scales of Psychological Well-being	63	0.74*
Short Depression Happiness scale	71	0.73*
WHO-5	79	0.77*
Life satisfaction		
Satisfaction with Life Scale	79	0.73*
Global Life Satisfaction	77	0.53*
Emotional intelligence		
Emotional Intelligence Scale	67	0.48*

*p < 0.01

**Figure 4:**
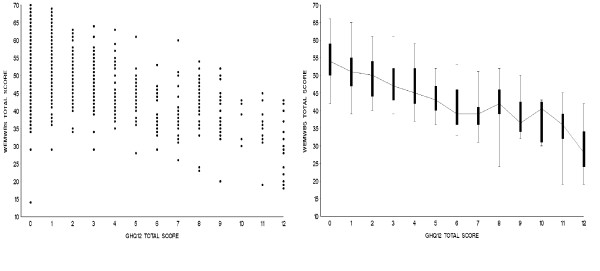
WEMWBS score vs. GHQ-12 score, scatter plot and box and 90% CI whisker plot: population sample.

The WEMWBS score showed a significant moderate sized negative correlation with mental ill-health, as represented by GHQ-12 score, in the population sample (r = -0.53, p < 0.01) using a Likert score, which persisted when a dichotomous scoring method, (with the four GHQ response categories being scored 0,0,1,1 [37]) was used (p < 0.01) (Figure 4).

In the population sample, the median WEMWBS score was significantly higher for men than for women (p < 0.05), as hypothesised (Table 3), and differences were also observed across age groups (p < 0.01), with higher scores observed in people aged 16–24 and 55–74. WEMWBS score was associated with higher socio-economic status as measured by both income levels and chief income earner social grade (both p < 0.01), with scores generally increasing as income or social grade increases.

**Table 3:** WEMWBS scores across demographic groups: population sample

**Variable**	**N**	**Median (95% CI)**	**p**
Total	1749	51 (51–52)	
Sex			
Male	783	52 (51–52)	< 0.05
Female	966	51 (50–52)	
Age in years			
16 – 24	176	53 (52–53)	< 0.01^KW^
25 – 34	245	51 (50–53)	
35 – 44	353	51 (49–52)	
45 – 54	306	50 (49–51)	
55 – 64	334	52 (51–53)	
65 – 74	274	52 (51–54)	
75+	61	51 (49–54)	
Tenure			
Own out right	523	52 (52–53)	< 0.01^KW^
Own with a mortgage	705	52 (51–52)	
Rent	519	50 (49–51)	
Self-perceived health status			
Very good	563	54 (54–55)	< 0.01^J^
Good	753	51 (51–52)	
Fair	319	47 (46–49)	
Poor	84	44 (40–46)	
Very poor	29	41 (36–47)	
Employment Status ^			
In work	968	52 (51–52)	< 0.01^KW^
Student	82	52 (50–54)	
Retired	465	51 (50–52)	
Unemployed	154	49 (47–51)	
Other	79	46 (43–50)	
Marital Status *			
Single	188	51 (49–53)	< 0.01^KW^
Married/Living as couple	418	52 (51–53)	
Widowed/Divorced/Separated	155	49 (46–51)	
Gross household income, pa *			
< £5000	55	48 (44–53)	< 0.01^J^
5000 – 14999	198	49 (47–51)	
15000 – 29999	180	53 (51–54)	
30000+	173	51 (49–53)	
Terminal Education Age *			
< 16	228	52 (50–53)	< 0.05^KW^
16 – 18	355	50 (49–51)	
19+	181	53 (51–54)	
Chief Income Earner Social Grade *			
A	38	55 (51–57)	< 0.01^J^
B	84	50 (48–53)	
C1	217	51 (50–53)	
C2	193	53 (51–54)	
D	101	50 (47–52)	
E	124	47 (44–51)	

* Tests conducted on a reduced set of patients. Variable only recorded in the HEPS survey.

95% CI = 95% confidence interval of the median

^KW ^= p-value generated from a Kruskal-Wallis test.

^J ^= p-value generated from a Jonckheere's tests for ordered alternatives.

^ = test conducted excluding the Other category

We also observed statistically significant differences between WEMWBS score and with housing tenure (p < 0.01) with higher scores among owner-occupiers. There were significant differences in WEMWBS scores across levels of marital status and employment status (both p < 0.01), with widowed, divorced or separated respondents and unemployed respondents reporting low scores. Significant differences were also observed with terminal age of education (p < 0.05), although confidence intervals overlapped for the < 16 and > 19 age groups. The highest levels of mental well-being were observed in those who had finished education at or older than 19 years of age (Table 3). This differs from the results of population mental health surveys [21].

### Test-retest reliability

Test-retest reliability at one week in the student sample was 0.83 (p < 0.01), indicating a high reliability for the new scale.

### Social desirability bias

Mean scores for the two sub-scales of the Balanced Inventory of Desirable Response (impression management and self-deception) were 6.7 (SD = 3.6) and 4.6 (SD = 3.2). respectively, in the student sample. Correlations with both the impression management and self-deception sub-scales were similar to, or lower than, other comparable scales and were lower than reported correlations with Affectometer 2 [16] (Table 4), which suggests that the new scale is not unduly susceptible to social desirability bias.

**Table 4:** Social desirability correlations for included scales: student sample

**Scale**	**N**	**Impression Management**	**Self-Deception**
Affectometer 2	115	-0.25**	0.55**
Global Life Satisfaction	62	0.26*	0.13
Satisfaction with Life scale	62	0.34**	0.40**
PANAS-PA	52	0.02	0.50**
PANAS-NA	51	0.03	-0.16
WEMWBS	115	0.18*	0.35**
WHO-5	62	-0.39**	-0.20

*p < 0.05

**p < 0.01

## Discussion

The new 14-item scale appears to have good face validity, as it covers the majority of the range of concepts associated with positive mental health, including both hedonic and eudaimonic aspects, positive affect, satisfying interpersonal relationships and positive functioning. WEMWBS performs well against accepted criteria at a population level. Unlike other commonly-used measures of mental health, WEMWBS did not show a ceiling effect in either of the study populations, indicating that the measure may have potential for documenting overall improvements in population mental well-being. The scale appears to have good content validity: response rates were high in both samples, although lower in the population sample than in the student sample. Confirmatory factor analysis supported the hypothesised one-factor solution, suggesting that WEMWBS measures a single underlying concept.

The internal consistency of the scale was high in both samples and only fell below a level of 0.8 once six items had been deleted, suggesting some redundancy in the scale. This may point to opportunities to reduce the length of the scale still further.

WEWMBS appears to be less prone to social desirability bias than other comparable scales assessed in this study. The correlation between overall score and the impression management sub-scale of the BIDR was lower than for any of the other scales tested, with the exception of the positive and negative sub-scales of the PANAS, although WEMWBS was more prone to self-deception bias than four of the other scales tested (SWLS, WHO-5, PANAS-NA and GLS). However it out-performed Affectometer 2 on both scales. This finding also needs to be reproduced in a population sample.

This study has a number of limitations. Whilst consensus is growing around many components of mental well-being there is still debate about the relevance of some concepts, for example spirituality and purpose in life. As WEMWBS was developed to enable monitoring of population health, it was considered important to cover only items which were likely to receive endorsement from the general UK population as related to mental well-being. Items relating to spirituality were therefore not included. The scale may need modification in the future to accommodate expansion of general population knowledge and understanding relating to the core components of mental well-being.

Although many of the tests for validity that were carried out on the initial student sample were repeated with a more robust population sample, space constraints meant that it was not possible to include all eight scales used to test the criterion validity of WEMWBS in this stage of the research. The results from the student sample suggest that WEWMBS shares common features with scales such as WHO-5, the Short-Depression Happiness Scale, Satisfaction with Life Scale and Scales of Psychological Well-being. The single-item measure of life satisfaction and the Emotional Intelligence Scale showed lower correlations, suggesting that WEMWBS may be measuring a different concept. However, these findings may not be generalisable to a wider population, given the limited age-range and other characteristics of the student sample. Similarly, it was only possible to assess the scale's test-retest reliability on the student sample and at an interval of one week. Further research is needed to identify whether this result is reproducible in a population sample and to test the stability of the scale over a longer period of time. In addition, the scale's capacity to detect changes in mental well-being at both individual and population-levels, for example after a significant life event or intervention, has not yet been assessed. This will be an important step in evaluating the scale's suitability for use in evaluation studies using a longitudinal design.

## Conclusion

WEMWBS shows high levels of internal consistency and reliability against accepted criteria. Short, acceptable and meaningful to general population groups, and relatively unsusceptible to bias, it is capable of distinguishing between different population groups in a way that is consistent with other population surveys. While the scale is likely to appeal to those evaluating mental health promotion initiatives (because of its positive focus), further research is needed to ensure that the scale is sensitive to change. The possibility that the scale could be shortened further also needs exploration. In the meanwhile, the scale's strong psychometric performance and lack of ceiling effects suggests that it is suitable for use in measuring mental well-being at a population level.

## Abbreviations

BIDR Balanced Inventory of Desired Responding.

EIS Emotional Intelligence Scale

EQ-5D VAS EuroQol Health Status Visual Analogue Scale

GHQ-12 General Health Questionnaire

GLS Global Life Satisfaction

HEPS Health Education Population Survey

SDHS Short Depression Happiness Scale

SPWB Scale of Psychological Well-Being

SWLS Satisfaction with Life Scale

PANAS Positive And Negative Affect Scale

PANAS-PA Positive And Negative Affect Scale – positive sub-scale

PANAS-NA Positive And Negative Affect Scale – negative sub-scale

WHO-5 WHO Wellbeing Index

WEMWBS Warwick-Edinburgh Mental Well-being Scale

## Competing interests

This research was commissioned by NHS Health Scotland.

## Authors' contributions

RT designed and coordinated the study, carried out statistical analyses and drafted the manuscript.

LH carried out all statistical analyses on population datasets and helped to draft the manuscript.

RF collected data, coordinated fieldwork and carried out qualitative and statistical analyses.

SP participated in the design and coordination of the study, coordinated fieldwork and helped to draft the manuscript.

SJ participated in the design and coordination of the study, advised on statistical analyses and helped to draft the manuscript.

SW participated in the coordination of the study and helped to draft the manuscript.

JP commissioned the study, participated in its design and coordination and helped to draft the manuscript.

JS advised on qualitative analyses and participated in the coordination of the study. 

SS-B conceived of the study, coordinated the development of the instrument and helped to draft the manuscript.

All authors read and approved the final manuscript.

## Supplementary Material

Additional file 1 The Warwick-Edinburgh Mental Well-being Scale. The fourteen-item Warwick-Edinburgh Mental Well-being Scale. Click here for file
