# Supplementary material for: The Warwick-Edinburgh Mental Well-being Scale (WEMWBS): development and UK validation
Source: Health Qual Life Outcomes. 2007 Nov 27;5:63. doi: 10.1186/1477-7525-5-63 (PMC2222612; doi:10.1186/1477-7525-5-63)
Supplement: Additional file 1 — The Warwick-Edinburgh Mental Well-being Scale. The fourteen-item Warwick-Edinburgh Mental Well-being Scale. [file 1477-7525-5-63-S1.pdf]

The Warwick-Edinburgh Mental Well-being Scale

(WEMWBS)

Below are some statements about feelings and thoughts.

Please tick the box that best describes your experience of each over the last 2 weeks

STATEMENTS	None of the time	Rarely	Some of the time	Often	All of the time
I've been feeling optimistic about the future	1	2	3	4	5
I've been feeling useful	1	2	3	4	5
I've been feeling relaxed	1	2	3	4	5
I've been feeling interested in other people	1	2	3	4	5
I've had energy to spare	1	2	3	4	5
I've been dealing with problems well	1	2	3	4	5
I've been thinking clearly	1	2	3	4	5
I've been feeling good about myself	1	2	3	4	5
I've been feeling close to other people	1	2	3	4	5
I've been feeling confident	1	2	3	4	5
I've been able to make up my own mind about things	1	2	3	4	5
I've been feeling loved	1	2	3	4	5
I've been interested in new things	1	2	3	4	5
I've been feeling cheerful	1	2	3	4	5

"Warwick Edinburgh Mental Well-Being Scale (WEMWBS)
© NHS Health Scotland, University of Warwick and University of Edinburgh, 2006,
all rights reserved."
